# Experimental datasets from mechanical testing and characterization of composites filled with PVCs waste

**DOI:** 10.1016/j.dib.2025.111894

**Published:** 2025-07-18

**Authors:** Ariyana Dwiputra Nugraha, Edi Purnomo, Fachri Rozi Afandi, Heru Wijayanto, Nugroho Karya Yudha, Eko Supriyanto, Alvin Dio Nugroho, Muhammad Akhsin Muflikhun

**Affiliations:** a PLN Research Institute, Indonesia; b Mechannical and Industrial Engineering Department, Universitas Gadjah Mada, Indonesia; c WIKA Energy, Indonesia; d Vytautas Magnus University, Lithuania; e Center for Energy Studies (PSE), Universitas Gadjah Mada, Indonesia

**Keywords:** Photovoltaic, Composite, Filler, Waste material, Manufacturing, Solar cell

## Abstract

This paper reports an investigation into composites containing photovoltaic (PVC) waste as a filler, offering a detailed set of data based on mechanical testing, material characterization, and simulation. The fabrication and testing of specimens followed established standards: ASTM D638 for tensile testing, ASTM D790 for flexural testing, ASTM D2240 for hardness, and ASTM D695 for compression tests. To ensure accuracy, the dimensions of the specimens were measured at three distinct points. The tensile tests were simulated using Abaqus 2023. The distribution of PVC waste as a composite filler can be observed using SEM/EDS, and FTIR analysis is used to identify the compounds present in the specimen. The data generated above provides information that the best ultimate tensile strength was obtained from the 4 % PVCs variation at 51.43 MPa, followed by a flexural strength of 45.54 MPa and a compressive strength of 35.38 MPa. The results of the SEM/EDS analysis indicate that the PVCs are primarily composed of carbon and silica. Additionally, a simulation utilising the Abaqus application was performed, revealing a discrepancy of <2 %. This data can be utilised by professionals in the field for further studies or to develop composites that incorporate PVC waste as a filler.

Specifications Table

**Table:** 

Subject	Engineering & Materials science
Specific subject area	Characterization, Mechanical Properties, Simulation, PVCs Filler
Type of data	Tables and Figures
Data collection	The mechanical parameters of tensile, compressive, and flexural strength of the material are derived from test results obtained using a Universal Testing Machine (UTM). The unprocessed data acquired from UTM includes the load and elongation of the material. Additionally, the raw data is transformed into stress and strain curves for tensile, compressive, and bending testing. The hardness of the composite was evaluated using the Shore D method, comparing its hardness to variations in PVC fillers. This study employed a simulation approach with Abaqus 2023 software for the tensile test. Characterization of composite materials with SEM/EDS and FTIR techniques.
Data source location	The data were obtained from advanced manufacturing lab, departement of mechanical and industrial engineering, Universitas Gadjah Mada, Indonesia.
Data accessibility	The data are available here and in data Mendeley data repository at this link https://data.mendeley.com/datasets/mpc7xpmc69/1
Related research article	Characteristics and evaluation of recycled waste PVCs as a filler in composite structures: Validation through simulation and experimental methods Articles related to these experimental datasets are available at this link https://doi.org/10.1016/j.jcomc.2024.100525

## 1 Value of the Data

• The data provided in this study relates to the tensile, compressive, bending, and hardness mechanical properties of composites with varying weight percentages of filler from recycled PVC waste.
• This research also provides insights into the distribution and surface of composites filled with PVC waste using SEM/EDS.
• Data on the characterization of functional groups or compound identification in the specimens provided in this study include FTIR analysis.
• Mechanical testing on this data can be used as a reference for researchers on how to use or utilize PVC waste as a filler in composites, where the composite is made using molds that are then filled with a mixture of resin, hardener, and PVC waste filler.

## 2 Background

The increasing global use of solar energy presents significant environmental challenges related to waste from e-waste i.e. lithium-ion battery [1,2], alkaline battery [3], PCBs [4], and solar panels that have reached the end of their lifespan, where the discarded materials are often not reused [5,6]. As a sustainable solution that supports the circular economy [7], recycling and reusing these materials as integrated system [8,9], especially as reinforcements in composites, the background of this research is an effort to address the waste problem generated by used photovoltaic cells (PVC) [[10], [11], [12]]. This is also supported by research that has used PVCs, such as the study by Chen, which used them as a battery mixture, resulting in an efficiency increase to 848.9 mA h *g* − 1 [13]. The outcome of Kokul's and Bhowmik's research with silicon crystals obtained from recycled solar panels is an enhancement of the modulus strength by 10 % [14]. In Palaniyappan's research with PVC waste material, there was an enhancement of the tensile test, flexural test, and compressive test of mechanical properties with an optimal weight percentage of 6 % [15]. Based on these promising findings, data collection through experiments was conducted to evaluate how PVC waste is used as a filler in composites, focusing on the utilization of PVC waste [16,17]. On the other hand, research conducted on PVC is also used to explore its potential as a filler in composites. The research technique employed encompasses experiments and simulations to evaluate the efficacy of PVC as a composite filler by quantifying tensile, compressive, and flexural strength, in addition to its characterization. The research has the potential to enrich the literature on PVC waste utilization, provide eco-friendly solutions, and facilitate new material creation through waste repurposing. In addition, this research not only contributes to the development of more efficient new materials but also supports environmental conservation efforts through waste reduction and the reuse of recycled materials, as well as further applications in wind turbines, structures, lightweight laminates, automotive structures, and sports equipment.

## 3 Data Description

**Fig. 1:**
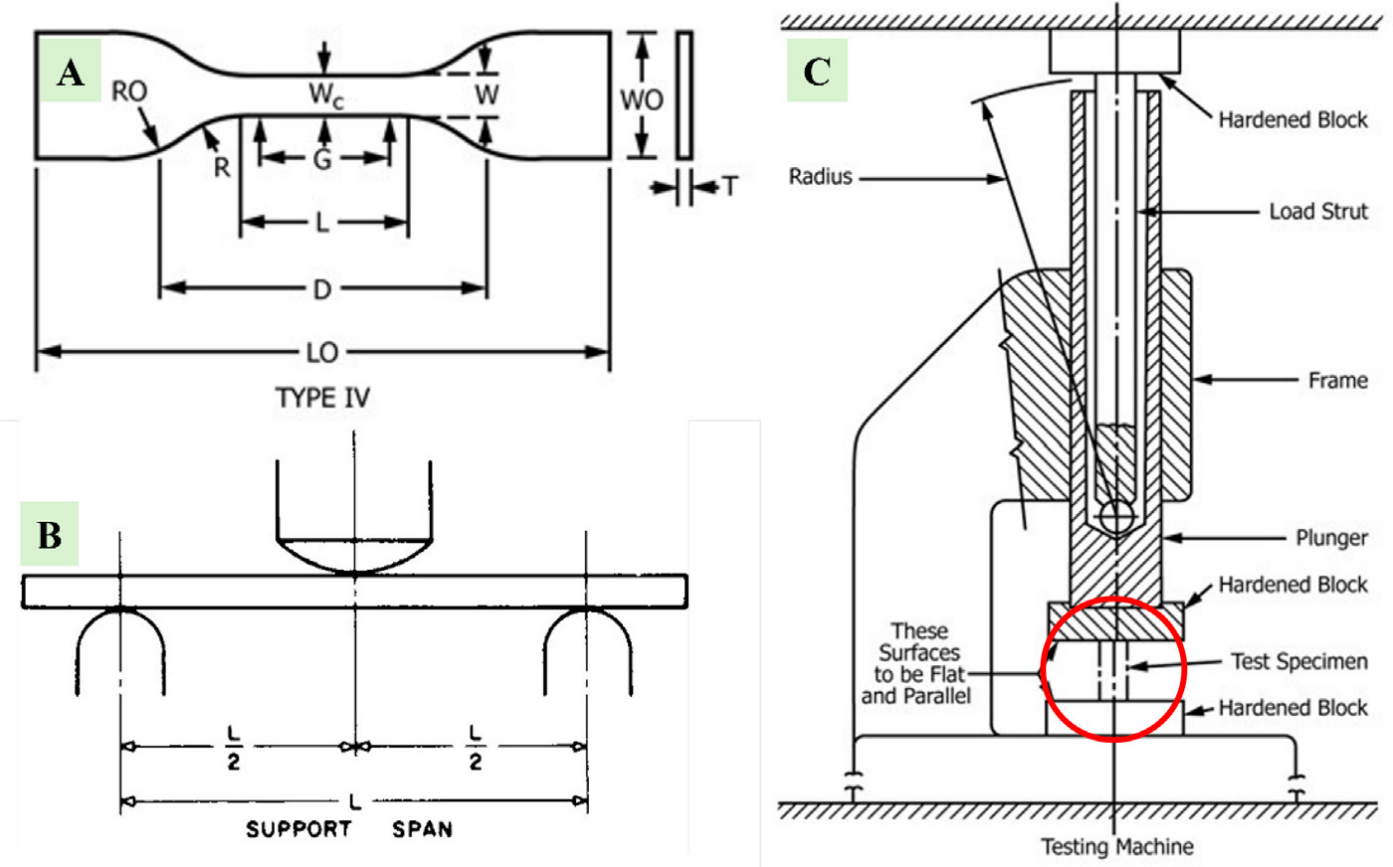
(A) Dog bone shape for tensile test, (B) Plate shape for bending test, and (C) Cylindrical shape for compression test [[19], [20], [21]].

**Fig. 2:**
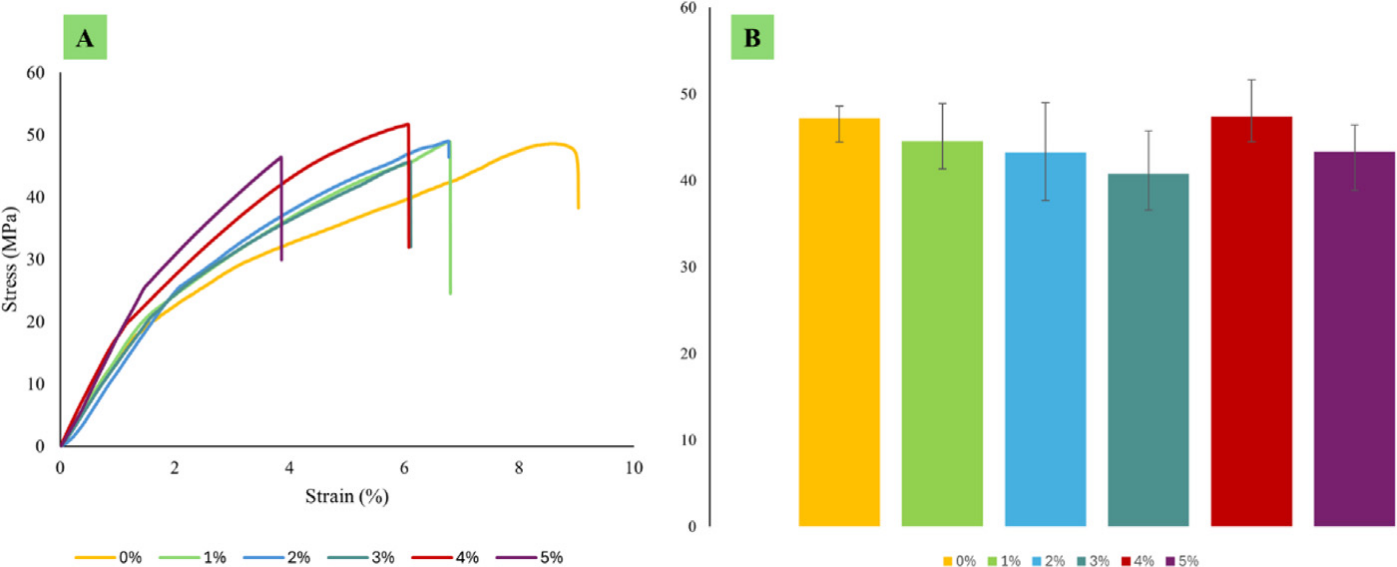
(A) Tensile curve stress-strain, (B) Comparison of tensile strength.

**Fig. 3:**
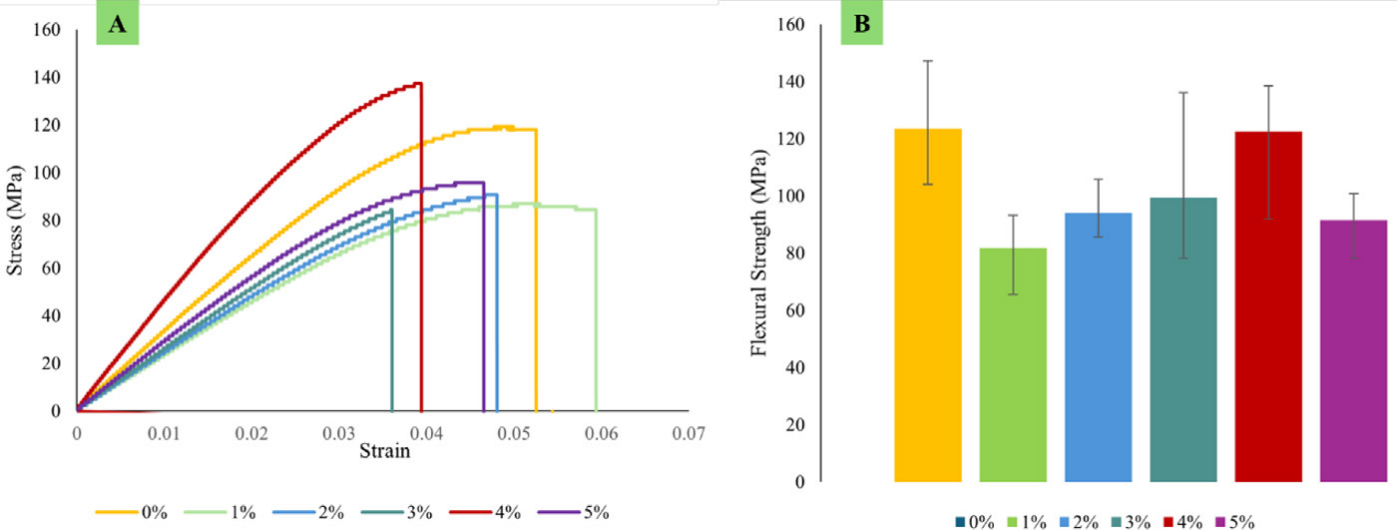
(A) Flexural curve stress-strain, (B) Comparison of flexural strength.

**Fig. 4:**
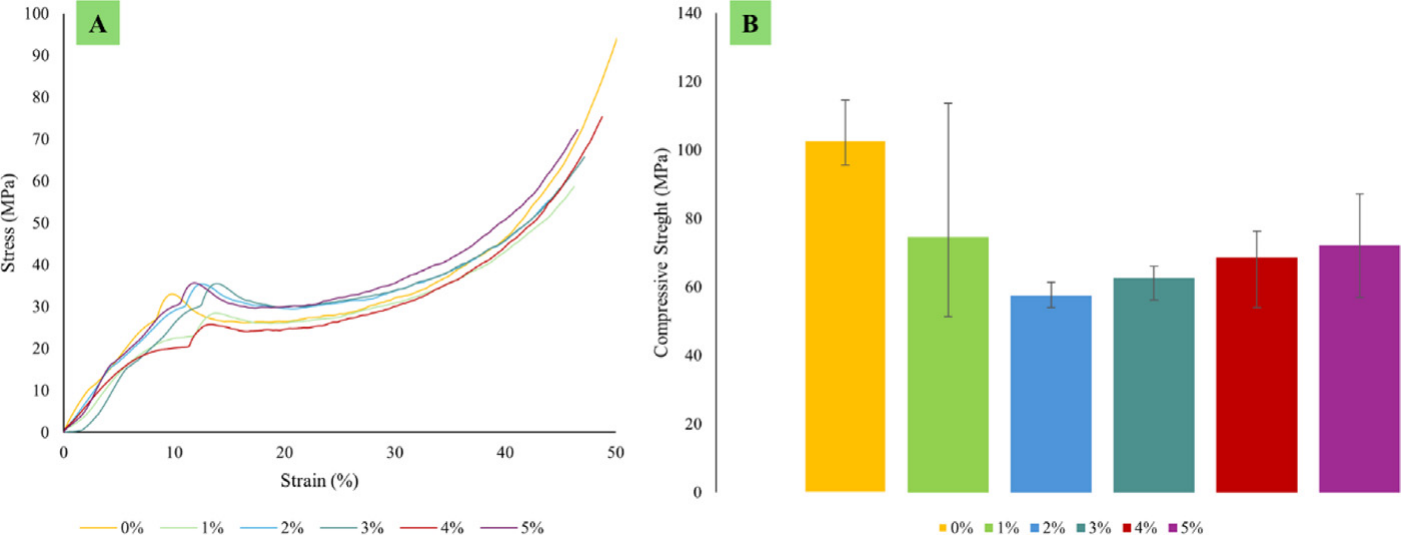
(A) Compression curve stress-strain, (B) Comparison of compression strength.

**Fig. 5:**
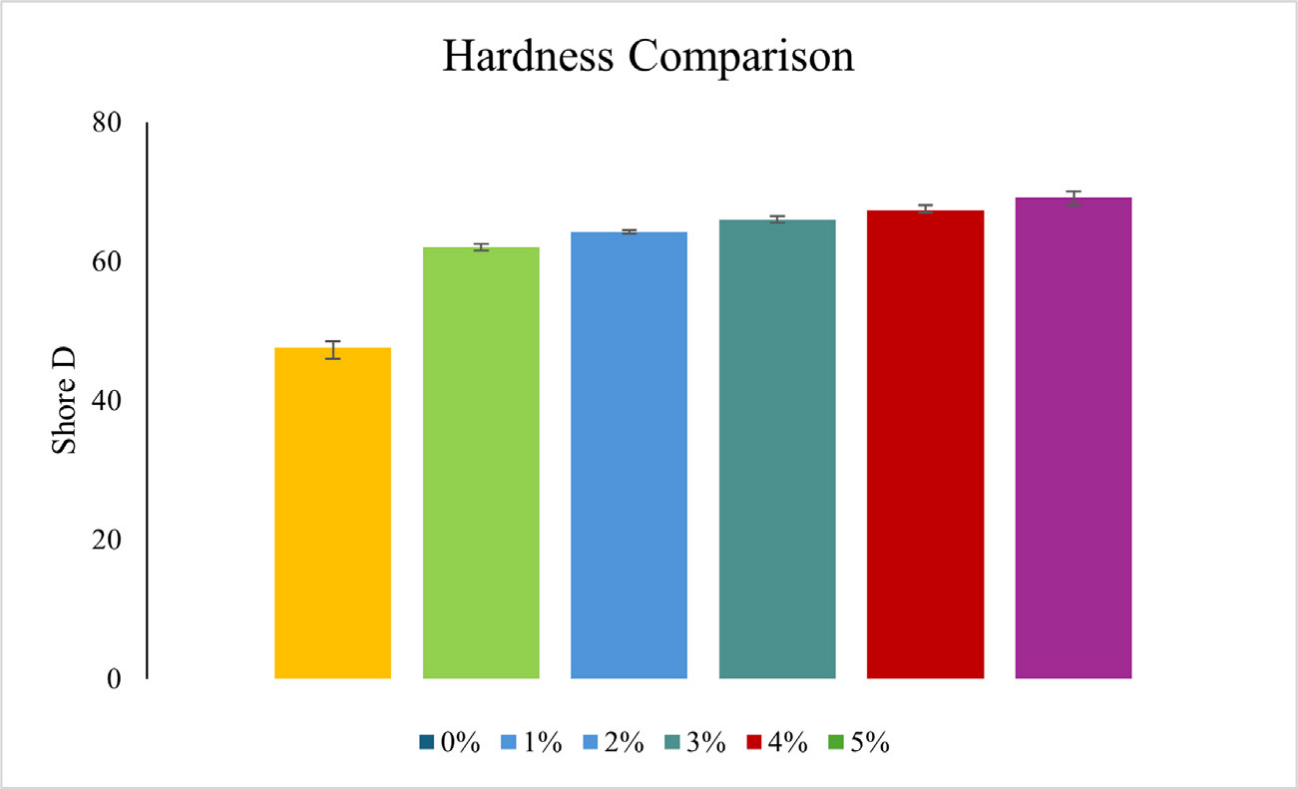
Comparison of hardness for each specimen variation.

**Fig. 6:**
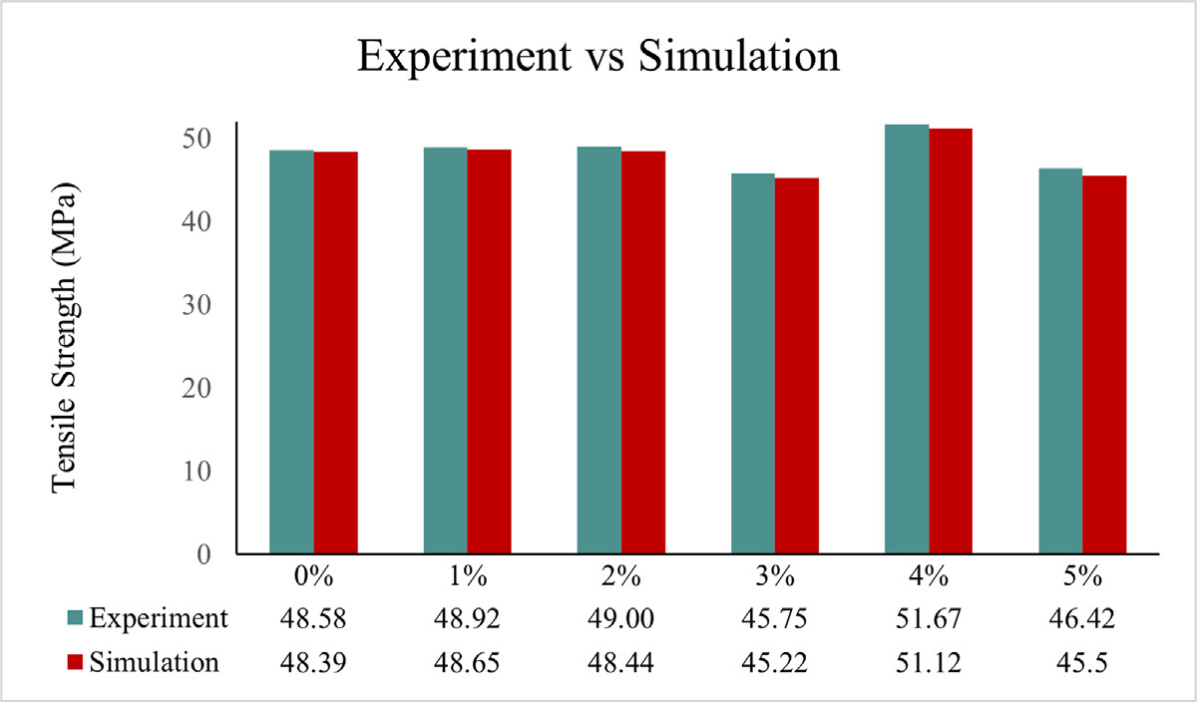
Comparison of testing and tensile test simulation.

Each type of composite test conducted in this study requires three specimens per variation, as detailed in Table 1. Next, the dimensions used in this study are in accordance with the standards shown in Table 2, and the specimen shape can be seen in Fig. 1. Raw load-displacement data were collected from the UTM machine and then turned into graphs showing stress versus strain, along with comparisons of tensile, flexural, and compressive strength, which are displayed in Fig. 2; Fig. 3; and Fig. 4, respectively. The comparative data from each mechanical test is taken as the average of 3 specimens for each variation. For the composite hardness test, refer to Fig. 5. The next step is to compare the tensile strength results obtained from direct tensile mechanical testing using UTM and simulations using ABAQUS 2023, refer to Fig. 6.

**Table 1:** List of required specimens.

No	Testing	Standard	Variation ( %)	Number of Spescimens
1.	Tensile Test	ASTM D638	0, 1, 2, 3, 4, 5	15
2.	Flexural Test	ASTM D790	0, 1, 2, 3, 4, 5	15
3.	Compression test	ASTM D695	0, 1, 2, 3, 4, 5	15
4.	Hardness Test	ASTM D2240	0, 1, 2, 3, 4, 5	5
5.	SEM	-	0, 1, 2, 3, 4, 5	5
6.	FTIR	-	0, 1, 2, 3, 4, 5	5
Total of Specimens				45

**Table 2:** Dimensions of the mechanical testing specimen.

No	Testing	Dimension			
1	Tensile Test	Gage Legth = 33 mm	Width = 6 mm	Thickness = 4 mm	Cross-section area = 24 mm^2^
2	Flexural Test	Support Span = 50.4 mm	Width = 12 mm	Thickness = 3.15 mm	Cross-section area = 37.8 mm^2^
3	Compression Test	Length = 25.6 mm	Diameter = 12.7	-	Cross-section area = 126.61 mm^2^

The specimens employed in material characterisation techniques, such as SEM and FTIR, are adequate for utilization in mechanical tests, including tensile testing and others [18]. Furthermore, the identical specimen may also be utilized for hardness testing.

**Fig. 7:**
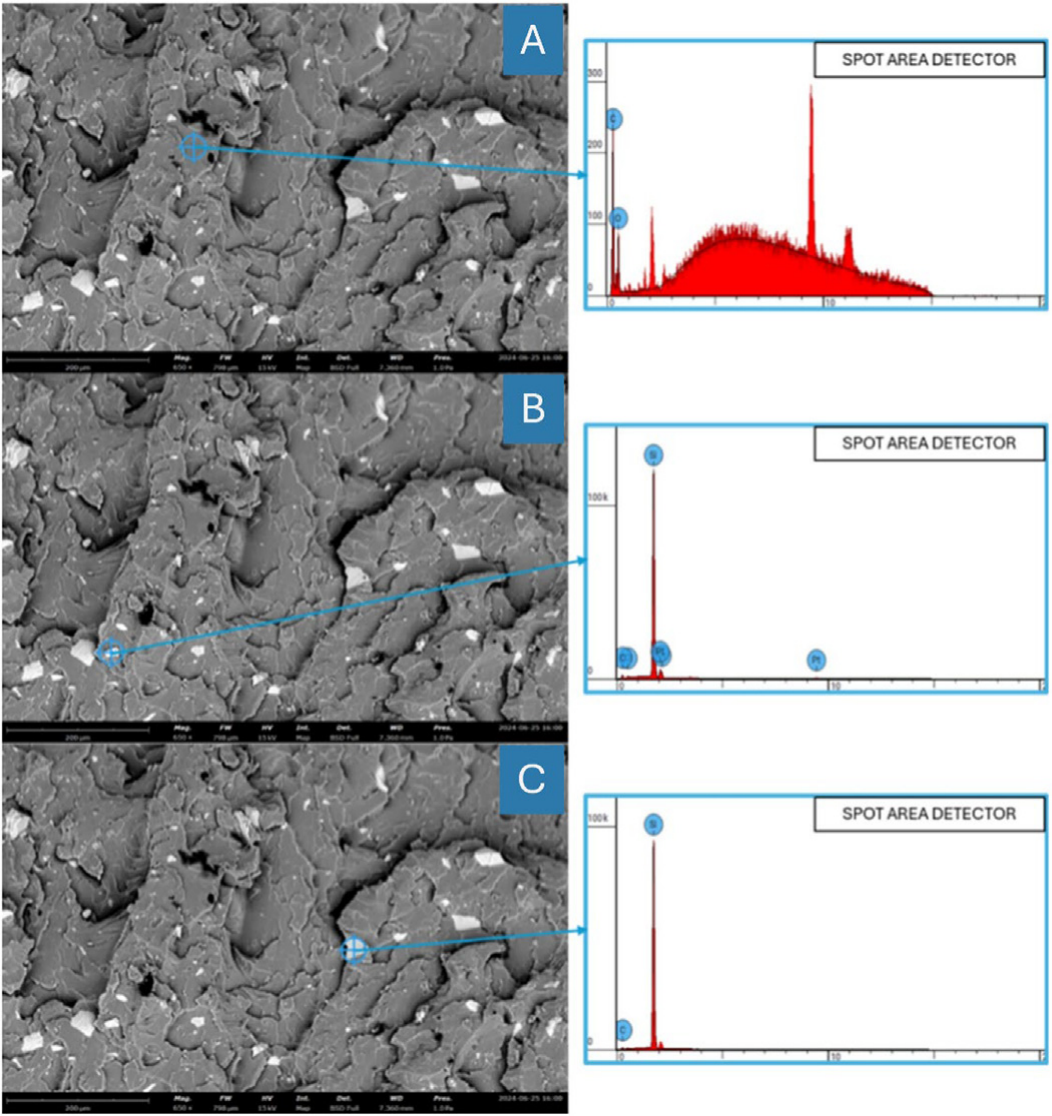
(A) SEM/EDS of resin in composite, (B) SEM/EDS of PVCs filler in composite, (C) SEM/EDS of PVCs filler in composite.

**Fig. 8:**
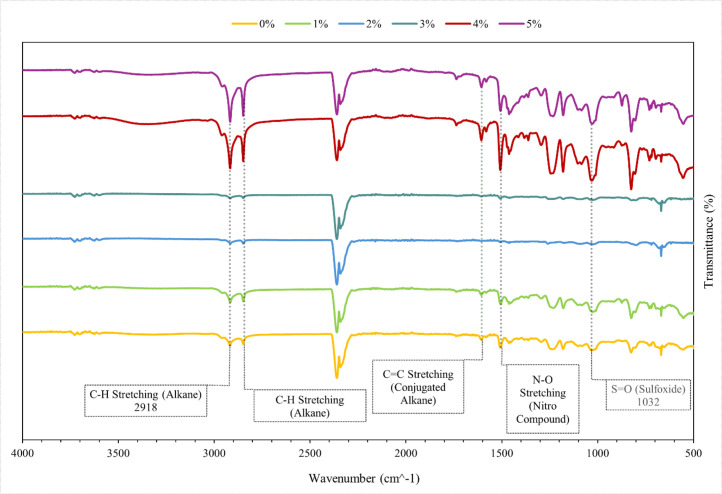
FTIR analysis of each variation of composite specimens.

This work essentially reveals the morphological differences between the dark matrix surface and the light PVC waste surface by means of SEM/EDS characterization. Three spot detection analysis at one site revealed that silica (Si) and carbon (C) made up the composite mostly. This study shows that the constituents influencing the mechanical strength increase of the material are silica and carbon. The surface and elemental distribution presented in the subsequent Table 3 and Fig. 7. FTIR analysis in this study focuses on identifying the compound bonds present with each addition of PVC waste filler into the composite. The experimental results showed several peak variations. The variation in intensity indicates that it can affect the polymerization process as well as the material's ability to withstand the load applied to the composite. The FTIR analysis is illustrated in Fig. 8.

**Table 3:** SEM/EDS result on a composite material with PVCs filler.

Specimen	Element Number	Element Symbol	Atomic Conc. ( %)	Weight Conc. ( %)
A	6	C	58.90	65.626
	8	O	41.10	34.374
B	6	C	65.6	58.9
	8	O	34.4	41.1
	14	Si	45.954	35.165
	17	Pt	5.295	7.113
C	6	C	15.30	29.701
	14	Si	84.7	70.299

## 4 Experimental Design, Materials and Methods

The use of powder as a composite filler is a measure aimed at enhancing its mechanical properties [[22], [23], [24]]. The challenge involved in using powder as a composite filler is determining the optimal proportion or percent of powder to use to enhance its mechanical properties. This study attempts to ascertain the optimal percentage of PVC powder waste that can improve the mechanical properties of the composite without filler by conducting trials with varying percentages of additional powder. The other subsequent problem in enhancing the mechanical strength of the composite using fillers is the uniform distribution of the powder within the matrix to avert stress concentration and premature failure. To prevent this problem, the composite material is blended with a mixer for a designated period.

### 4.1 Mechanical test

Composite filler in this study using solar panels produced by PT. Wijaya Karya Industri Energi (Persero) Tbk, which have been crushed and standardized to a size of 38 µm using sieving. The preparation of PVC waste particles involves the hand crushing of used solar cells. Subsequently, it is subjected to sieving through mesh sizes of 60, 150, 300, and ultimately 400. The mean particle size generated is roughly 38 µm. The matrix used is bisphenol A-epichlorohydrin epoxy resin and cycloaliphatic amine as the epoxy hardener, both of which are products of PT Justus Kimia Raya, Indonesia. Next, the composite manufacturing process is carried out by mixing PVC waste powder with resin and hardener, where the resin to hardener ratio is 2:1. The mixing process of the three materials takes five minutes using the Thinky Are-301 Planetary Mixer. The mixing process with that equipment facilitates uniform distribution of the filler throughout the matrix. The result of the mixing process is poured into molds that have been prepared from silicone material. The variation in filler mixing into the composite matrix is 0–5 %. The curing process of the composite is carried out for eight hours, after which it will be subjected to mechanical testing.

Mechanical testing on the specimens consists of tensile testing using the ASTM D638 standard [19], hardness testing using the ASTM D2240 standard [25], flexural testing using the ASTM D790 standard [20], and compressive testing using the ASTM D695 standard [21].During the mechanical testing process, the UTM used is the Universal Testing Machine Carson CRN-50 [26]. The calculation for the mechanical testing of composite specimens reinforced with PVC waste filler is done using the equation in Table 4, involving load and elongation obtained from the UTM.

**Table 4:** The equation to calculate stregth, stress, and strain of material.

Specimen Testing	Material Stregth (Mpa)	Stress (Mpa)	Strain
Tensile	$\sigma_{uts}=\;\frac{F_{max}}{A}$	$\sigma =\;\frac{F}{A}$	$\varepsilon =\frac{\Delta L}{L0}$
Compressive	$\sigma_{c}=\;\frac{F}{A}$	$\sigma =\;\frac{F}{A}$	$\varepsilon =\frac{\Delta L}{L0}$
Flexural	$\sigma_{f}=\;\frac{3.F.L}{2.b.d^{2}}$	$\sigma =\;\frac{M.c}{I}$	$\varepsilon =\frac{6.P.L}{W.t^{2}}$

### 4.2 Tensile simulation

During the simulation phase, it is crucial to acquire tensile property data from each specimen variation, as each data point will be utilized to delineate the qualities required for the simulation process. The tensile strength outcomes from the simulation will thereafter be compared with the results of the direct tensile test.

### 4.3 Characterization

The results of the composite manufacturing process described above will then be followed by specimen preparation according to the sample holder for SEM/EDS analysis. The Phenom™ ProX Desktop SEM is the product used to observe the distribution and surface of the specimen. SEM/EDS will later provide results on the surface morphology and distribution of PVC waste in the composite reinforced by PVC waste.

## Limitations

• The experimental data is applicable solely to composites composed of the identical material. The composite material comprises epoxy resin and hardener in a 2:1 ratio, to which PVC waste with an average particle size of 38 µm is incorporated. Subsequently, pour the resultant mixture into the mould.
• A crucial factor to consider in this research for data acquisition is the fluctuation in the percentage of PVC waste, which must be maintained at an optimal level, neither excessively high nor excessively low. If a standard deviation is identified during data processing, it is advisable to augment the number of sample tests to mitigate the standard deviation.

## Ethics Statement

The current work does not involve human subject, animal experiment, or any data collected from social media platforms.

## CRediT Author Statement

**Ariyana Dwiputra Nugraha:** Formal Analysis, Writing - Original Draft, Writing - Review & Editing. **Edi Purnomo:** Conceptualization, Methodology, Formal Analysis, Writing - Original Draft, Writing -Review & Editing. **Fachri Rozi Afandi:** Writing - Review & Editing. **Heru Wijayanto:** Writing - Review & Editing. **Nugroho Karya Yudha:** Writing - Review & Editing. **Eko Supriyanto:** Writing - Review & Editing. **Alvin Dio Nugroho:** Writing - Review & Editing. **Muhammad Akhsin Muflikhun:** Conceptualization, Methodology, Writing - Original Draft, Writing Review & Editing, Supervision, Project administration, Funding acquisition

## Data Availability

Mendeley DataRaw data of Composite with PVCs Filler (Original data)
